# Hepatic Cyst: An Unusual Suspect of Syncope

**DOI:** 10.1155/2020/1659718

**Published:** 2020-02-28

**Authors:** Mohammad K. Choudhry, Bei Xiong, Antony Anandaraj, John Trillo

**Affiliations:** ^1^Department of Gastroenterology, NYCHH-Coney Island Hospital, Brooklyn, NY, USA; ^2^Department of Medicine, NYCHHC-Coney Island Hospital, Brooklyn, NY, USA

## Abstract

The patient is a 75-year-old man with history of diabetes and hypertension who presented with syncope after experiencing sharp, 10/10 right flank and abdominal pain worsening over three weeks associated with decreased appetite. Physical examination revealed hepatomegaly and right lower quadrant (RUQ) tenderness, negative for peritoneal signs. Bloodwork showed leukocytosis (13 K/mcl), alkaline phosphatase (141 U/L), total bilirubin (2.0 mg/dL), and gamma-glutamyl transferase (172 U/L). Computed Tomography (CT) revealed multiple hepatic cysts with the largest measuring 17 × 14 × 18 cm (Figure 1). Parenteral opiates provided minimal relief. Cardiac and neurologic etiologies of syncope were ruled out. The patient's course was complicated by opioid-induced delirium as his abdominal pain progressively worsened despite escalating doses of parenteral and oral analgesics. Gastroenterology and interventional radiology consulted to evaluate for Glisson's capsular stretch. Therapeutic aspiration yielded 2.5 L of serous fluid, which alleviated the patient's pain. Cytology was negative for malignancy. Opiates were titrated down. Repeat CT (Figure 2) showed cysts that were significantly reduced in size. The patient showed complete resolution of symptoms and was subsequently discharged. We present a rare case of a large hepatic cyst causing syncope. In the appropriate clinical setting, syncope with RUQ tenderness and hepatomegaly should raise the index of suspicion for hepatic cysts.

## 1. Background

Syncope is transient loss of consciousness with many potential causes. The evaluation of syncope typically involves a thorough history and physical exam followed by diagnostic studies to rule out possible etiologies including orthostatic hypotension, cardiac arrhythmia, structural heart diseases, neurologic, mediation side effects, psychiatric, and stress-related conditions. We present a 75-year-old male with severe pain-related vasovagal syncope due to a hepatic cyst.

## 2. Case Presentation

The patient is a 75-year-old man with a past medical history of type 2 diabetes mellitus and hypertension who was brought in by emergency medical services after experiencing a possible syncope due to severe right side flank pain. The patient had two witnessed syncopal episodes while waiting for the ambulance service as per the wife. He was then admitted for further evaluation of syncope.

The patient had worsening right-sided flank pain since the past 3 weeks. He described the pain as sharp, 10/10 in intensity which was also associated with a decrease in appetite. He had never experienced these symptoms in the past and denied any associated nausea, vomiting, dysuria, hematuria, fever, chills, or rigors.

**Figure 1:**
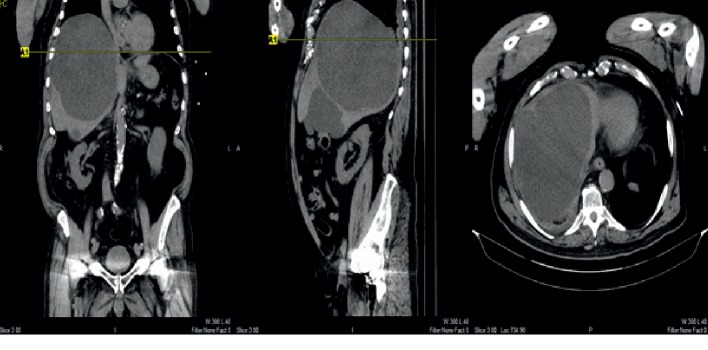
Initial CT abdomen/pelvis with multiple views of large hepatic cysts.

On examination, he was alert and oriented to time, place, and person complaining of abdominal pain. His abdomen was soft and distended, and right lower quadrant was tender to palpate without any guarding. He also had significant hepatomegaly. Laboratory findings were indicative of leukocytosis (13), elevated alkaline phosphatase (141), total bilirubin (2.0), and GGT (172). CT head ruled out any intracranial pathologies. CT abdomen/pelvis (Figure 1) showed multiple hepatic cysts. Largest cyst was in the right lobe measuring about 17 × 14 × 18 cm and others measured up to 9 cm and 4 cm in the right inferior lobe and the left lobe, respectively. Since admission, he was started on parenteral opiates which minimally helped his pain symptoms with no further reported syncopal episodes. Additional testing including EEG, 24 hours holter monitor, and echocardiogram did not reveal any cardiac or neurologic etiologies of syncope.

During his course, his abdominal pain symptoms got worse despite escalating doses of parenteral and oral opioid pain medication, which eventually progressed to opiate induced delirium. Gastroenterology as well as interventional radiology was consulted since his symptoms were attributed to stretching of the liver capsule. A multidisciplinary decision was made after a detailed discussion about the risk of reaccumulation with the patient, and a therapeutic aspiration of the cyst was performed. 2.5 L of serous colored fluid was aspirated, and cytology was negative for malignancy. This intervention resolved his pain symptoms and opiates were titrated down as tolerated over the next few days.

**Figure 2:**
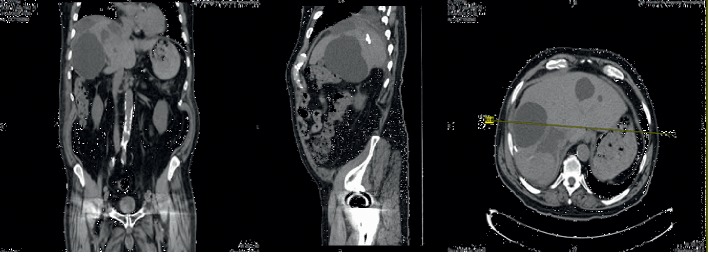
Postdrainage CT abdomen/pelvis showing significant reduction of the size of the cysts.

A Repeat abdominal/Pelvis CT (Figure 2) showed a significantly smaller cyst measuring 8 × 6 × 6 cm. In an attempt to prevent reaccumulation, sclerotherapy was offered to the patient and family, but due to the risk of iatrogenic sclerosis of the biliary system, the patient refused sclerotherapy at the time. His abdominal pain eventually resolved requiring no pain medications with significant improvement in his appetite and was discharged to the rehabilitation center.

## 3. Discussion

This case demonstrates a unique presentation of hepatic simple cyst-induced vasovagal syncope. Most liver cysts are incidental findings on imaging usually requiring no treatment and tend to have a benign course [6]. Our patient presented with a huge 17 × 14 × 18 cm cyst with capsular stretching that led to severe pain-related vasovagal syncope due to parasympathetic activation. Even though aspiration of the cyst is not the typical intervention given the high likelihood of reaccumulation, we proceeded with the goal of therapeutic pain relief. This intervention of therapeutic CT-guided aspiration not only provided resolution of symptoms also helped with rapid taper of his opioid pain medications. Simple aspiration has a high likelihood of recurrence (10–25 percent recurrence rate), and sclerotherapy was considered in this patient to prevent reaccumulation; however, due to the high incidence of iatrogenic sclerosis, sclerotherapy was not performed. In our patient, *R* factor was suggestive of cholestasis; however clinical and radiological findings were not supportive of cholestatic injury.

Benign liver cysts occur in 4–5% of individuals with a female predominance [1]. They are usually incidental findings on imaging because no more than 10–15% of these patients have symptoms. Prevalence of simple cysts occurs more commonly in the right lobe and increases with age. More than half of individuals older than 60 years of age are likely to have one or more simple cysts. Cysts that reach giant sizes occur almost exclusively in patients greater than 50 years of age [2].

Larger cysts can lead to complications including hemorrhage, pain, rupture into the peritoneal cavity, infection, and obstruction of the biliary duct [3]. Therapeutic interventions include needle aspiration with or without injection of sclerosing agents (alcohol and minocycline hydrochloride), laparoscopic unroofing of the cyst, and liver resection [4]. It is recommended that interval (6 to 12 months) ultrasounds be utilized to monitor for reaccumulation, growth, or resolution of the hepatic cyst [5].
